# Association of body mass index and maternal age with first stage duration of labour

**DOI:** 10.1038/s41598-021-93217-5

**Published:** 2021-07-05

**Authors:** Louise Lundborg, Xingrong Liu, Katarina Åberg, Anna Sandström, Ellen L. Tilden, Olof Stephansson, Mia Ahlberg

**Affiliations:** 1 Clinical Epidemiology Division, Department of Medicine, Solna, Karolinska Institutet, Stockholm, Sweden; 2 Division of Obstetrics and Gynecology, Department of Women’s and Children’s Health, Karolinska University Hospital, Stockholm, Sweden; 3 Department of Nurse-Midwifery and Department of Obstetrics and Gynecology, Oregon Health & Science University School of Medicine, Portland, OR USA; 4 Department of Obstetrics and Gynecology, Oregon Health & Science University School of Medicine, Portland, OR USA

**Keywords:** Medical research, Epidemiology

## Abstract

To evaluate associations between early-pregnancy body mass index (BMI) and active first stage labour duration, accounting for possible interaction with maternal age, we conducted a cohort study of women with spontaneous onset of labour allocated to Robson group 1. Quantile regression analysis was performed to estimate first stage labour duration between BMI categories in two maternal age subgroups (more and less than 30 years). Results show that obesity (BMI > 30) among younger women (< 30 years) increased the median labour duration of first stage by 30 min compared with normal weight women (BMI < 25), and time difference estimated at the 90th quantile was more than 1 h. Active first stage labour time differences between obese and normal weight women was modified by maternal age. In conclusion: (a) obesity is associated with longer duration of first stage of labour, and (b) maternal age is an effect modifier for this association. This novel finding of an effect modification between BMI and maternal age contributes to the body of evidence that supports a more individualized approach when describing labour duration.

## Introduction

Duration of labour varies between and within populations^1–3^. Determining normal and abnormal labour duration is complex because duration is influenced by multiple factors, such as: spontaneous or induced start of labour^4,5^, parity^2^, obstetrical management^6–9^, infant and maternal anthropometrics such as body mass index (BMI) and maternal age^10–17^. During the past 40 years the proportion of Swedish women giving birth to their first child at 35 years of age or greater increased from 5 to 20%. Importantly, the proportion of Swedish women with obesity (BMI ≥ 30) has almost doubled between 1992 and 2014, the prevalence of maternal obesity was 11% in year 2014^18^. By 2025, estimates indicate that 21% of Swedish women will be severely obese (BMI > 35)^19^. These trends of rising age at first delivery in tandem with rising rates of obesity are obstetric challenges^20,21^.

It is well established that obese women have increased risk of labour induction, labour dystocia (prolonged labour duration), instrumental vaginal delivery and caesarean delivery^8,14,17,22–25^. The mechanism of a possible synergetic effect of obesity and age has not been evaluated in studies on labour duration with spontaneous onset^26,27^. Advanced maternal age is a risk factor for pregnancy-related complications, and the age-related decline of uterine performance is an important contributor to the increased risk of labour dystocia and caesarean delivery^13,28–30^. Studies on the association between maternal age and first stage labour duration show inconsistent results. Zaki et al.^13^ found that increasing maternal age decreased duration of first stage labour, but Greenberg et al.^10^ did not find such association. Our purpose in designing this study was to provide estimate of the magnitude for the effect of both BMI and age on increasing durations. We recognized that prior investigations had focused predominantly on measures of tendency, therefore our intent was to use quantile regression to capture trends of widespread labour durations. Our hypothesis was that BMI are associated with longer labour durations and that this relationship is modified by age.

The objective of this study was to determine the association between early-pregnancy BMI and labour duration by distinct quantiles and to investigate if this is modified by maternal age.

## Methods

### Study design and population

**Figure 1:**
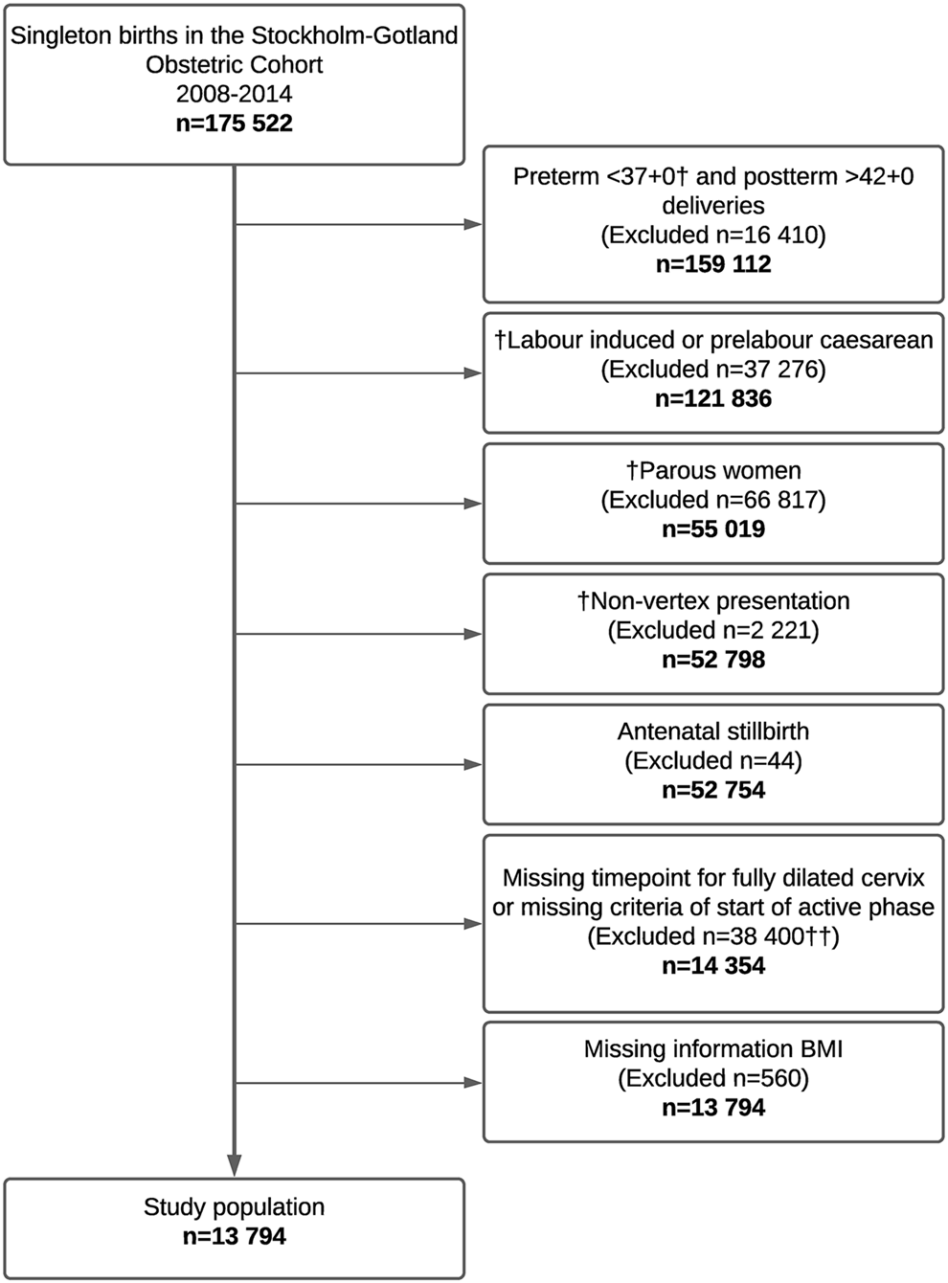
Flowchart of cohort selection. ^†^Conditions indicated to fulfill the Robson 1 criteria. ^††^Including 557 women with caesarean delivery in first stage. *n* corresponds to numbers left *after* exclusions. Women in latent labour at admission were not included in the final study population since they did not fulfil the definition of start active phase. *BMI* body mass index.

We conducted a cohort study of singleton live births among nulliparous women using data from the regional population-based Stockholm-Gotland Obstetric Cohort. The Swedish Ethical Review Authority (Etikprövningsmyndigheten), Sweden approved the study and all procedures performed were in accordance with the ethical standards of the committee. This database includes all deliveries in the seven delivery units in the Stockholm-Gotland region, which corresponds to 25% of all pregnancies and deliveries (n = 175,522) in Sweden during the study period (January 2008 to October 2014) It includes information on maternal characteristics including maternal medical history, antenatal and obstetrical care from pregnancy throughout birth and neonatal care. Standardized recorded variables and prospectively collected data captures information directly from the electronic medical record system using the person-unique national registration numbers of mothers and infants^31^. Maternity care is free of charge and offered to all women in Sweden. The first visit generally occurs between 7 and 12 weeks of gestation, with approximately 10–12 visits during pregnancy, depending on parity. The seven delivery units in the region are comparable and share guidelines for obstetrical management. Based on the Robson Ten-Group Classification system^32,33^, data in this study was extracted for women allocated to Robson group 1: nulliparous, term gestations (≤ 37 + 0 weeks) with vertex, singleton pregnancies who experienced spontaneous labour onset and with a live foetus (n = 13,794). Figure 1 presents a flow chart of the study population. Women were further excluded due to missingness related to: (a) early pregnancy BMI (n = 560), (b) start of active labour or completed cervical dilation (n = 38,400). Women with caesarean delivery during first stage were excluded.

### Exposure

The main exposure was early-pregnancy body mass index (BMI). Maternal early-pregnancy weight and height were measured at the first antenatal care visit, with BMI calculated as the ratio of weight (in kilograms) divided by the square of height (in meters). Early-pregnancy BMI was categorized into normal weight (< 25 kg/m^2^), overweight (≥ 25 to 29.9), and obesity (≥ 30.0).

### Outcomes

The primary outcome was duration of first stage of labour. The scope of the study was the active phase of first stage of labour, i.e. the progressing period of the first stage, whereas the latent phase of labour duration and women with a cervical dilation > 5 cm at admission were not considered in this study. Onset of first stage of labour was defined by four strict hierarchy criteria (C1–C4) to identify the correct timepoint for customizing the start of the active first stage for each woman. Criteria one (C1) corresponded to cervical effacement (complete or nearly 100% effacement), dilation of 3 to 4 cm and painful contractions; (C2) cervical effacement, dilation of 3 to 4 cm and spontaneous rupture of membranes; (C3) cervical effacement, regular painful contractions and dilation at admission < 4 cm; and (C4) cervical effacement, regular painful contractions, and dilation of 5 cm. If labour assessment did not meet the first criteria, subsequent criteria were considered to identify the correct timepoint for women´s individual onset of first stage of labour. The parameters included in these criteria (i.e. cervical dilation, rupture of membranes, contractions) are standardly used as guidelines across Sweden to identify women in active first stage of labour for admission to the obstetric units and documented by the attending midwife^34^. The end of the first stage of active labour was defined by the time-point of the first notation that the cervix was fully dilated. The secondary outcome was total duration of active labour, with onset defined as the time that active labour began through second stage and termination defined as the time of birth. Both outcomes were analysed as continuous variables and labour duration prior to hospital admission was not considered in this study.

### Covariates

Information on maternal characteristics such as age, height, weight, BMI, smoking status and co-habitation status were retrieved from data collected at the first antenatal visit. Gestational age was determined using the following hierarchy: (a) embryo transfer, (b) first trimester ultrasound, (c) early second trimester ultrasound offered to all women, (d) date of last menstrual period, and (e) postnatal assessment. Onset of labour, antenatal stillbirth, time of cervix dilation, mode of delivery, use of epidural analgesia and oxytocin were obtained from the standardized delivery records. Maternal age, maternal height, smoking status in early pregnancy, co-habitation status (co-habitation is considered being a proxy for continuous support^35^), and year of birth were identified as confounders by a directed acyclic graph (see DAG, Supplementary Fig. S1) Maternal age was also considered a potential effect modifier for the association between BMI and labour duration. Birthweight, hypertensive disease (gestational hypertension and preeclampsia), gestational diabetes, use of epidural and oxytocin were considered to be mediators as they lie on the pathway between the exposure and outcomes (see DAG, Supplementary Fig. S1).

### Statistical analysis

Descriptive statistics of baseline characteristics were stratified by two maternal age groups (younger: < 30 and older: ≥ 30 years) and three BMI-categories (normal weight: ≤ 25, overweight: 25–29.9 and obese: ≥ 30 kg/m^2^) (Table 1). The distribution of main and secondary outcomes at different quantiles (q10–q90) were calculated for each stratum of the three BMI groups in the total study population and subsequent (after interaction test was found statistically significant) stratified by maternal age group (Table 2). The absolute time difference between the BMI categories was calculated, with the normal-weight group as reference. The overall differences in distributions of the outcomes between BMI-categories were tested using the non-parametric Kolmogorov–Smirnov test, with the normal-weight group as the reference (see Supplementary Fig. S2).

**Table 1:** Baseline characteristics for the study population 13,794 women.

	Term nulliparous women with spontaneous labour onset					
	Age < 30			Age ≥ 30		
	BMI < 25 (n = 5292)	BMI = 25–29.9 (n = 1 451)	BMI = 30+ (n = 541)	BMI < 25 (n = 4 913)	BMI = 25–29.9 (n = 1 229)	BMI = 30+ (n = 368)
**Mother's characteristic**						
Age, years, mean (SD)	25.4 (3.0)	25.1 (3.0)	25.0 (2.9)	33.2 (2.8)	33.5 (3.0)	34.0 (3.2)
Early-pregnancy BMI, kg/m^2^, mean	21.6	26.9	33.7	21.7	26.9	33.4
Maternal height, cm, mean (SD)	165.5 (6.4)	164.9 (6.4)	164.8 (7.0)	170.0 (6.3)	165.8 (6.7)	166.0 (6.8)
Gestational age in days, mean (SD)	281 (7.5)	281.7 (7.5)	281.4 (7.7)	281.9 (7.5)	281.8 (7.6)	282.2 (7.4)
Family situation						
Single	7.18%	9.4%	7.8%	4.3%	5.8%	6.0%
Co-habitant	89.4%	87.5%	87.8%	92.0%	90.9%	89.7%
Unknown	3.38%	3.2%	4.4%	3.7%	3.3%	4.6%
Smoking status						
Non-smoker	93.1%	92.0%	87.3%	97.3%	95.4%	94.3%
Smoker	6.1%	7.6%	12.4%	2.1%	4.1%	4.6%
Not known	0.9%	0.4%	0.4%	0.7%	0.6%	1.1%
Pregestational diabetes	0.4%	0.7%	1.5%	0.2%	0.9%	1.9%
Essential hypertension	3.0%	4.5%	5.9%	3.6%	5.9%	9.8%
**Characteristics related to labour**						
Oxytocin augmentation	49.6%	51.8%	59.5%	52.6%	56.5%	56.8%
Epidural analgesia	67.9%	66.7%	71.4%	64.6%	63.5%	65.2%
Mode of birth						
Spontaneous vaginal	78.3%	75.5%	75.1%	67.9%	63.5%	64.7%
Operative vaginal	15.3%	14.3%	12.9%	21.0%	20.8%	17.7%
Caesarean delivery (second stage)	6.4%	10.3%	12.0%	11.1%	15.8%	17.7%
Duration of second stage (min), median (IQR)	85 (46–146)	84 (45–143)	77 (40–141)	105 (57–178)	106 (57–179)	97 (49–167)
Fetal position at birth						
Anterior	95.0%	93.3%	94.1%	93.6%	92.8%	92.7%
Posterior	5.0%	6.8%	5.9%	6.4%	7.2%	7.3%
**Characteristics of newborn**						
Birthweight (gram), mean (SD)	3472 (434)	3564 (453)	3616 (469)	3 488 (433)	3573 (452)	3625 (490)
Year of birth						
2008–2010	38.2%	36.9%	34.6%	38.5%	38.0%	39.1%
2011–2014	61.8%	63.1%	65.4%	61.5%	62.0%	60.9%

**Table 2:** Distribution of active phase and totallabour duration in each of three BMI-categories, in the total study population and stratified by age groups (< 30 vs ≥ 30).

	Maternal BMI (kg/m^2^)	N	Distribution of duration of active first stage of labour (in hours) by quantiles [0–100%, intervals of 10]									Distribution of total duration of active labour [active first stage and second stage] (in hours) by quantiles [0–100%, intervals of 10]								
			q10	q20	q30	q40	q50	q60	q70	q80	q90	q10	q20	q30	q40	q50	q60	q70	q80	q90
Total study population	< 25	10,205	1.97	3.08	4.04	5.00	5.97	7.00	8.08	9.50	11.75	3.17	4.58	5.78	6.82	7.87	9.02	10.28	11.82	14.23
	25–29.9	2680	2.04	3.17	4.15	5.10	6.09	7.15	8.37	9.82	11.97	3.27	4.72	5.83	6.95	7.98	9.27	10.54	12.15	14.47
	≥ 30	909	2.03	3.17	4.14	5.19	6.32	7.50	8.75	10.23	12.68	3.28	4.53	5.76	6.94	8.05	9.13	10.75	12.57	15.02
Younger age group (< 30 years)	< 25	5292	1.90	3.00	3.92	4.79	5.67	6.67	7.68	9.10	11.23	3.03	4.30	5.38	6.50	7.45	8.48	9.70	11.22	13.63
	25–29.9	1451	2.00	3.10	3.95	4.87	5.78	6.75	7.97	9.50	11.53	3.12	4.45	5.47	6.63	7.48	8.70	9.98	11.40	13.77
	30+	541	1.92	3.05	4.08	5.08	6.17	7.40	8.48	10.08	12.95	3.17	4.43	5.57	6.55	7.80	8.88	10.53	12.45	15.28
Older age group (≥ 30 years)	< 25	4913	2.00	3.20	4.23	5.25	6.25	7.40	8.47	9.97	12.25	3.38	4.94	6.17	7.25	8.40	9.62	10.90	12.35	14.83
	25–29.9	1229	2.08	3.29	4.48	5.35	6.45	7.68	8.93	10.17	12.39	3.59	5.20	6.27	7.38	8.80	9.95	11.24	13.07	14.98
	30+	368	2.15	3.27	4.20	5.45	6.40	7.70	9.02	10.39	12.42	3.65	4.90	6.25	7.38	8.27	9.71	10.89	12.70	14.79

Quantile regression was used to investigate associations of first stage labour duration with increasing BMI category in both univariate and multivariable analyses. Mean, median and time difference at distinct quantile levels, with 95% confidence intervals (CI), was calculated. The association between increasing BMI and outcome distributions were examined under the following three analytic approaches.

First, quantile regression models were used to examine the association between primary and secondary outcomes with increasing BMI category, while adjusting for maternal age, maternal height, smoking status, co-habitation status, and year of birth in multivariable analyses.

Subsequently, an interaction term between BMI and age was added into the quantile regression models to investigate whether the interaction term was statistically significant.

Finally, based on the observed time differences in the descriptive analysis and because interaction between BMI and age was demonstrated, we performed stratified sub-group analyses by age group, with adjustment for maternal height, smoking status, co-habitation status, and year of birth in multivariable analyses.

Data management and analyses were conducted using SAS version 9.4 {http://www.sas.com/} SAS institute, Cary, NC, USA) and R version 3.6.1 (R Foundation for Statistical Computing, Vienna, Austria, 2016). All statistical tests were 2-sided, with p-value of less than 0.05 as statistical significance.

### Ethical approval

Permission for this study was obtained from Swedish Ethical Review Authority (In Swedish: Etikprövningsmyndigheten, Sweden, http://etikprovningsmyndigheten.se), No 2009/275-31, 2012/365-32, 2013/792, 2014/177-32, 2014/962-32. Swedish Ethical Review Authority was previously (at the time for permission) named Regional Ethical Review board in Stockholm, (In Swedish: Regionala etikprövningsnämnden Stockholm), Sweden. In accordance with their decision, we did not obtain informed consent from participants in the study. Our research was performed in accordance with relevant guidelines and regulations. All data were de-identified prior to analysis, and the study was conducted in accordance with the STROBE guidelines for observational cohort studies.

## Results

Of the 175,522 observations in the Stockholm-Gotland Obstetric cohort 13,794 women meet the inclusion criteria during the study period (2008–2014), most exclusions were due to missing information on start or end of the active first stage (exposure (Fig. 1)). Detailed baseline characteristics for all women allocated to Robson 1 (Target population n = 52,754) is reported in Supp. Table S5 for comparison to the study cohort. Baseline characteristics are reported in Table 1, for the 13,794 women included in the study cohort and stratified by maternal age. The distribution (%) of early-pregnancy BMI (mean), gestational age, and birthweight were similar in both age strata. Compared to normal weight women, obese women were more likely to receive oxytocin for labour augmentation and to deliver via caesarean. In addition, caesarean delivery was more common among older obese women (older 17.7% vs. younger 12.0%). The duration of second stage of labour was longer among older women regardless of BMI category, see Table 1.

Table 2 show the distribution of duration of active first stage across the three BMI categories in the study population overall and stratified by age. When comparing all women in the cohort, duration of the active first stage increased with higher BMI.

The outcome distribution by quantile levels is presented in Table 3, with normal BMI as reference. Difference in distribution of duration in the total study population in the 90th quantile was 0.93 h. The corresponding difference in distribution of duration, 90th quantile, within the age stratified groups was 1.72 in the younger age group and 0.17 h in the older age group, Table 3. The median duration was 0.50 h longer for obese women compared to normal weight women in younger age group (6.17 h vs. 5.67 h, respectively), Table 2 and 3. Results from the Kolmogorov–Smirnov test revealed a statistically significant difference (p-value = 0.002), comparing obese to normal-weight, in the younger age stratum, for the primary outcome (see Supplementary Fig. S2). While similar patterns were noted among older women, differences were much less pronounced, Table 3. For the secondary outcome, total duration of labour (including both first stage active and second stage), older women with obesity had faster labours at several quantiles compared to older women with lower BMIs, Table 3. No significant differences were found from the Kolmogorov–Smirnov test among the older age stratum (see Supplementary Fig. S2). Multivariable regression estimates for the total study population for both primary and secondary outcomes were shown in Supplementary Tables S1 and S2, with univariate regression estimates presented in Supplementary Tables S3 and S4. Further, the interaction term was statistically significant (by Wald test) for both outcomes at larger quantile levels, respectively.

**Table 3:** Difference in labour duration at different quantile levels, comparing the obese, over- to normal-weight group based on the total study population and two age subgroups separately.

	Maternal BMI (kg/m^2^)	N	Difference in duration (hours) of *active first stage* in quantiles (hours)									Difference in *total duration* of active labour at below quantiles (hours)								
			q10	q20	q30	q40	q50	q60	q70	q80	q90	q10	q20	q30	q40	q50	q60	q70	q80	q90
Total study population	< 25 (reference)	10,205	0	0	0	0	0	0	0	0	0	0	0	0	0	0	0	0	0	0
	25–29.9	2680	0.07	0.08	0.11	0.10	0.13	0.15	0.28	0.32	0.22	0.10	0.13	0.05	0.13	0.12	0.25	0.26	0.33	0.23
	30+	909	0.07	0.08	0.10	0.19	**0.35**	**0.50**	**0.67**	**0.73**	**0.93**	0.11	− 0.06	− 0.03	0.12	**0.18**	**0.12**	**0.47**	**0.76**	**0.78**
Younger age group (< 30 years)	< 25 (reference)	5292	0	0	0	0	0	0	0	0	0	0	0	0	0	0	0	0	0	0
	25–29.9	1451	0.10	0.10	0.03	0.08	0.12	0.08	0.28	0.40	0.30	0.08	0.15	0.08	0.13	0.03	0.22	0.28	0.18	0.13
	30+	541	0.02	0.05	0.17	0.29	*0.50*	*0.73*	*0.80*	*0.99*	*1.72*	0.13	0.13	0.18	0.05	*0.35*	*0.40*	*0.83*	*1.23*	*1.65*
Older age group (≥ 30 years)	< 25 (reference)	4913	0	0	0	0	0	0	0	0	0	0	0	0	0	0	0	0	0	0
	25–29.9	1229	0.08	0.09	0.25	0.10	0.20	0.28	0.46	0.20	0.14	0.21	0.26	0.11	0.13	0.40	0.33	0.34	0.72	0.15
	30+	368	0.15	0.07	− 0.03	0.20	***0.15***	***0.30***	***0.55***	***0.42***	***0.17***	0.27	− 0.04	0.08	0.13	***− 0.13***	***0.09***	*** − 0.01***	***0.35***	*** − 0.04***

With respect to effect modification of maternal age we then performed multivariable analysis stratified by maternal age, Tables 4 and 5. The naïve linear regression analysis for the association of the mean of the outcome, was statistically significant for the younger obese women (mean difference 0.58 h, 95% CI 0.24–0.91, p-value ≤ 0.01; Table 4) for the primary outcome and secondary outcome (mean difference 0.51 h, 95% CI 0.13–0.89, p-value ≤ 0.05; Table 5). In the younger age group, obese women had a longer duration of active first stage than normal-weight women at higher quantile levels (estimated difference in duration of active first stage at the 90th quantile, 1.35 h, 95% CI 0.53–2.17; Table 4). Similarly, for the secondary outcome total duration of active labour, in the younger age group, the estimated difference at the 90th quantile level was 1.37 h (95% CI 0.69–2.06; Table 5). Comparisons showed no significant difference in labour duration in the older age group. In sensitivity analysis, when excluding women with caesarean delivery in second stage (9.8% of the study population), the estimates were only marginally altered (see Supplementary Table S5). Lastly, we examined distribution of exposures (BMI and age) among those excluded due to caesarean delivery (n = 557) during first stage compared to the study population. The median BMI among these women was 23.0 compared to 22.7 in the study population, and the median age was 31 and 29 years respectively.

**Table 4:** Multivariable regression analysis for association of duration of active first stage with maternal early-pregnancy BMI, using the Stockholm-Gotland obstetric database, 2008–2014.

	Maternal BMI (kg/m^2^)	Multivariable regression estimates: *difference in duration of active first stage*^a^ at mean or quantiles (95% confidence intervals, hours)									
		Linear regression	Quantile regression estimates								
		Mean	q10	q20	q30	q40	q50	q60	q70	q80	q90
Younger age group (< 30 years)	< 25 (reference)	0	0	0	0	0	0	0	0	0	0
	25–29.9	0.14 (− 0.08, 0.36)	0.08 (− 0.16, 0.33)	0.10 (− 0.09, 0.29)	0.02 (− 0.19, 0.23)	− 0.00 (− 0.25, 0.25)	− 0.03 (− 0.32, 0.27)	0.10 (− 0.19, 0.38)	0.20 (− 0.13, 0.53)	0.22 (− 0.20, 0.64)	0.329 (− 0.24, 0.82)
	30+	0.58** (0.24, 0.91)	0.08 (− 0.16, 0.31)	− 0.01 (− 0.28, 0.26)	0.12 (− 0.34, 0.57)	0.24 (− 0.14, 0.61)	0.49 (− 0.13, 1.12)	0.82** (0.41, 1.22)	0.72** (0.23, 1.20)	1.17** (0.50, 1.84)	1.35** (0.53, 2.17)
Older age group (≥ 30 years)	< 25 (reference)	0	0	0	0	0	0	0	0	0	0
	25–29.9	0.12 (− 0.13, 0.37)	0.05 (− 0.18, 0.28)	0.18 (− 0.12, 0.48)	0.23 (− 0.04, 0.51)	0.01 (− 0.28, 0.30)	0.14 (− 0.21, 0.49)	0.22 (− 0.15, 0.59)	0.26 (− 0.06, 0.59)	0.13 (− 0.24, 0.49)	0.07 (− 0.40, 0.54)
	30+	0.23 (− 0.20, 0.65)	0.12 (− 0.36, 0.60)	0.17 (− 0.20, 0.54)	0.09 (− 0.41, 0.58)	− 0.01 (− 0.64, 0.61)	0.31 (− 0.28, 0.89)	0.52 (− 0.11, 1.15)	0.67 (0.03, 1.32)	0.38 (− 0.22, 0.98)	0.38 (− 0.25, 1.00)

Multivariable regression analysis on two age sub-groups separately: maternal early pregnancy BMI categorized into three sub-groups, with adjustment for maternal height (in restricted cubic splines with 3 degrees of freedom), smoking status (dummy variable), co-habitation status (categorical variable), year of birth (categorical variable); stratified on two separate age groups.

^a^Duration of active first stage: the length from the start of active phase of labour until the time point of the cervix fully dilated.

**p-value < 0.01.

**Table 5:** Multivariable regression analysis for association of total duration of active labour with maternal early-pregnancy BMI, using the Stockholm-Gotland obstetric database, 2008–2014.

	Maternal BMI (kg/m^2^)	Multivariable regression estimates: *difference in total duration of active labour*^a^ at mean or quantiles (95% confidence intervals, hours)									
		Linear regression	Quantile regression estimates at below quantiles								
		Mean	q10	q20	q30	q40	q50	q60	q70	q80	q90
Younger age group (< 30 years)	< 25 (reference)	0	0	0	0	0	0	0	0	0	0
	25–29.9	0.13 (-0.12, 0.37)	0.05 (− 0.20, 0.29)	0.06 (− 0.18, 0.30)	0.00 (− 0.31, 0.30)	0.12 (− 0.18, 0.41)	0.02 (− 0.26, 0.30)	0.23 (− 0.12, 0.59)	0.19 (− 0.16, 0.54)	0.08 (− 0.29, 0.45)	0.19 (− 0.44, 0.82)
	30+	0.51* (0.13, 0.89)	0.04 (− 0.28, 0.35)	0.00 (− 0.45, 0.44)	− 0.01 (− 0.51, 0.49)	0.11 (− 0.34, 0.57)	0.47 (− 0.07, 1.01)	0.46 (− 0.07, 0.99)	0.70* (0.10, 1.30)	1.02* (0.08, 1.97)	1.37** (0.69, 2.06)
Older age group (≥ 30 years)	< 25 (reference)	0	0	0	0	0	0	0	0	0	0
	25–29.9	0.18 (− 0.10, 0.46)	0.06 (− 0.29, 0.40)	0.31 (0.01, 0.61)	0.02 (− 0.28, 0.32)	0.17 (− 0.21, 0.54)	0.20 (− 0.18, 0.57)	0.25 (− 0.14, 0.63)	0.21 (− 0.12, 0.54)	0.37 (− 0.08, 0.82)	0.23 (− 0.32, 0.78)
	30+	0.11 (− 0.36, 0.58)	0.35 (− 0.09, 0.79)	0.11 (− 0.29, 0.51)	0.07 (− 0.63, 0.76)	0.03 (− 0.45, 0.50)	− 0.20 (− 0.75, 0.35)	0.13 (− 0.69, 0.95)	0.43 (− 0.28, 1.14)	0.40 (-0.55, 1.35)	0.15 (− 0.59, 0.88)

Multivariable regression analysis on two age sub-groups separately: maternal early pregnancy BMI categorized into three sub-groups, with adjustment for maternal height (in restricted cubic splines with 3 degrees of freedom), smoking status (dummy variable), co-habitation status (categorical variable), year of birth (categorical variable); stratified on two separate age groups.

^a^Total duration of active labour: the length from the time point of the cervix fully dilated until birth.

*p-value < 0.05; **p-value < 0.01.

## Discussion

### Main findings

In this cohort study, we found that obesity was associated with increased duration of labour at higher quantiles and longer active first stage labour duration was especially pronounced among obese women younger than 30 years of age. Hence, the active first stage labour time differences between obese and normal weight women was modified by maternal age.

### Results and statistical approach implications

The adjusted age-stratified quantile regression analyses showed that the duration of active first stage of labour for the younger obese group was more than 1 h longer compared to the younger normal-weight group (at the 90th percentile). We noted a trend towards longer duration for women ≥ 30 years across BMI-categories; this trend was not statistically significant, which might be explained by lack of power in this study.

The rationale behind assessing effect modification was to identify if the association between BMI and labour duration is modified by maternal age. Our study complements the body of evidence on how maternal individual characteristics influence labour duration by presenting the association between BMI and labour duration stratified by age^36,37^.

In line with previous studies on duration and progression of active first stage, Norman et al.^23^ concluded that increasing BMI was associated with longer labour duration in nulliparous women, excluding women with a caesarean delivery before fully dilated cervix; however, this study did not take maternal age into account. Moreover, both Vahratian et al.^38^ and Kominiarek et al.^25^ presented similar results in studies on labour progression during first stage, using interval-censored regression analysis, the latter only including women who reached fully dilated cervix. In another Swedish cohort study based on nulliparous women with spontaneous onset of labour, Carlhall et al.^17^ found that total duration of active labour increased significantly with increasing BMI. Yet, for women within Robson group 1, Ellekjaer et al.^14^ reported that BMI was not significantly associated with total duration of active labour. However, these studies did not stratify the analysis by age, which is important since age according to the present study modify the association between BMI and labour duration. If a covariate is found to be an effect modifier, stratification triumphs adjusting since adjusting would take away the effect of a covariate instead of revealing the full effect of that variable^36,37^.=

Different approaches and measures have been used to investigate associations between BMI and labour duration, e.g. odds ratios for prolonged labour duration^17^, delivery rate ratios^39^, median difference and mean labour curves^23,25^. Compared to previous studies, time difference at distinct quantile levels between BMI-categories will add a clinically comprehensive measure with additional information for communicating labour progression and duration estimates.

Our choice to define 30 years of age as the threshold for stratification was informed by several studies^21,40^. Among nulliparous women with spontaneous onset of labour, Smith et al.^41^ demonstrated a trend of increasing duration with increasing maternal age which levelled out at 30 years of age.

Our results confirm seminal research on obese women, showing an increasing trend of augmentation with oxytocin and a correlation with increased duration of both first stage and total duration of active labour^22,42,43^. Use of oxytocin, epidural analgesia, instrumental delivery and caesarean delivery differed across BMI-categories and might impact labour duration. However, these variables were considered mediators and were therefore not adjusted for in the multivariable analyses^44^ (Suppl. Fig. S1).

### Strengths and limitations

There are several study strengths. In spontaneous labour, women are admitted to the hospital at different stages of labour which is an established challenge in studies evaluating labour duration. Selection bias could be introduced if the selection process allows women to be included in a study exclusively on the basis “admitted to the hospital”. Hereby, studies on labour duration are dependent on defining a correct timepoint for start of the active first stage. Therefore, to minimize the risk of including women in the latent phase, we applied a strict definition for start of active first stage labour to identify the timepoint for onset of labour, not exclusively based on dilation at admission. This hierarchy enabled us to find the start for each woman, which is important when using first stage duration as the exposure of interest. This definition included several clinically validated parameters beyond cervical dilation (i.e. rupture of membranes, contractions) and was used in combination with the Robson classification system, which we consider a strength. During the study period, no change was made to clinical guidelines with respect to active phase definition. Women admitted to the delivery hospitals between 2008 and 2014 in the region who met the described definition were deemed in the active first stage of labour and managed accordingly. The baseline characteristics for both study population and the target population, Supplementary Table S5, comparable to the previous study by Lundborg et al.^1^, showed that the subgroup of women in this study was equivalent to the population at large in terms of key characteristics. Further, this study cohort is based on a population with a large proportion of spontaneous vaginal births which is of importance when evaluating first stage duration and progression. Identifying women according to the Robson classification system enables extended understanding for drivers (labour dystocia, maternal anthropometrics, clinical management and decision making) for caesarean delivery and facilitates comparative data in diverse populations^33^. The Robson classification is a validated system supported by World Health Organization to both inform and improve maternity care^33^. Selection bias is further minimized as all pregnant women in Sweden are offered standardized health care from pregnancy to postpartum care free of charge^31^.

This study is not without limitations, unmeasured confounders and residual confounding cannot be ruled out, e.g. the information of maternal socio-economic status was not recorded in this database and potentially impacted on maternal age at first child. Another challenge in research on labour duration is how to avoid selection bias related to factors such as caesarean delivery during first stage of labour. In this study, women with missing time-point for fully dilated cervix (i.e. those with a caesarean delivery in first stage) were excluded from the analysis, a previously adopted method in seminal studies^25,45^. However, the exclusion of caesarean deliveries during first stage is not likely to impact our study findings as those women only constituted 1.1% of the target population (Fig. 1, Suppl. Table S6).

The study cohort was restricted to women allocated to Robson classification group 1 which limit the generalisability to other Robson groups. The non-significant results for obese women > 30 years of age might be explained by a higher number of inductions in this group. Older nulliparous women more often have co-morbidity leading to inductions and guidelines in the study region recommend older first-time mothers (> 40 years) induction at gestational week 41 + 0. This left us with a reduced number of women ≥ 40 years of age with spontaneous onset of labour limiting the statistical inference and robustness of the results in this age group. Studies of women with induction of labour is an important direction for future research.

### Clinical implications

Obese women allocated to Robson group 1 was experiencing longer labour duration than normal weight women, and it was particularly pronounced for women with longer first stage labour durations. When stratifying on age we could identify significantly longer durations among the younger obese age group (< 30 years) but not among obese women older than 30 years. The non-significant results among obese women older than 30 years is possibly due to lack of power. Hence, these results inform both clinicians and obese women with spontaneous onset that labour might last longer compared with normal weight women even in younger ages.

Labour duration norms were previously established using measures of central tendency. This approach has been challenged by contemporary labour progress research and may not be clinically meaningful for identifying women with a prolonged labour duration. Moreover, labouring women may differ in important ways that shape labour progress. Both maternal BMI and age are available information at onset of labour for clinicians which could provide greater clinical insight into anticipated labour progression and guide prospective intrapartum management during labour.

## Conclusions

The duration of active first stage of labour and total labour duration from start of active phase to birth was increased in Robson group 1 women with obesity and was modified by age. By stratifying by age longer labour durations were found showing that age is a modifier rather than a confounder. These novel findings of an effect modification between BMI and maternal age contributes to the body of evidence that supports a more individualized approach in defining labour duration. Considered with findings of other research, these results indicate a need for a revised definition of normal and prolonged labour where different maternal characteristics are considered, including maternal age and BMI.

## Supplementary Information

Supplementary Information.

## Data Availability

The Stockholm-Gotland Obstetric Cohort was used for this study. Information in the databased was retrieved from the medical record system Obstetrix. The database is stored in the Unit of Clinical epidemiology at Karolinska Institutet Stockholm, Sweden. Public data sharing from this database is not permitted. However, any research can access the data by obtaining an ethical approval from a regional ethical review board and thereafter contacting the Unit of Clinical epidemiology. Department of Medicine, Karolinska Institutet, Professor sven.cnattingius@ki.se for obtaining the original data.
