# Supplementary material for: Association of body mass index and maternal age with first stage duration of labour
Source: Sci Rep. 2021 Jul 5;11:13843. doi: 10.1038/s41598-021-93217-5 (PMC8257589; doi:10.1038/s41598-021-93217-5)
Supplement: Supplementary file 1 — Supplementary Information. [file 41598_2021_93217_MOESM1_ESM.pdf]

Association of Body Mass Index and maternal age with first stage duration of labour

Louise Lundborg[1*], Xingrong Liu[1,] Katarina Åberg[1], Anna Sandström[1,2,4], Ellen L. Tilden[3,4], Olof Stephansson[1,2], Mia Ahlberg[1,2]

[1] Clinical Epidemiology Division, Department of Medicine, Solna, Karolinska Institutet, Stockholm, Sweden.

[2] Department of Women´s and Children´s Health, Division of Obstetrics and Gynecology, Karolinska University Hospital, Stockholm.

[3] Department of Nurse-Midwifery and Department of Obstetrics and Gynecology, Oregon Health & Science University Schools of Nursing and Medicine, Portland, Oregon

[4] Department of Obstetrics and Gynecology, Oregon Health & Science University School of Medicine, Portland, Oregon

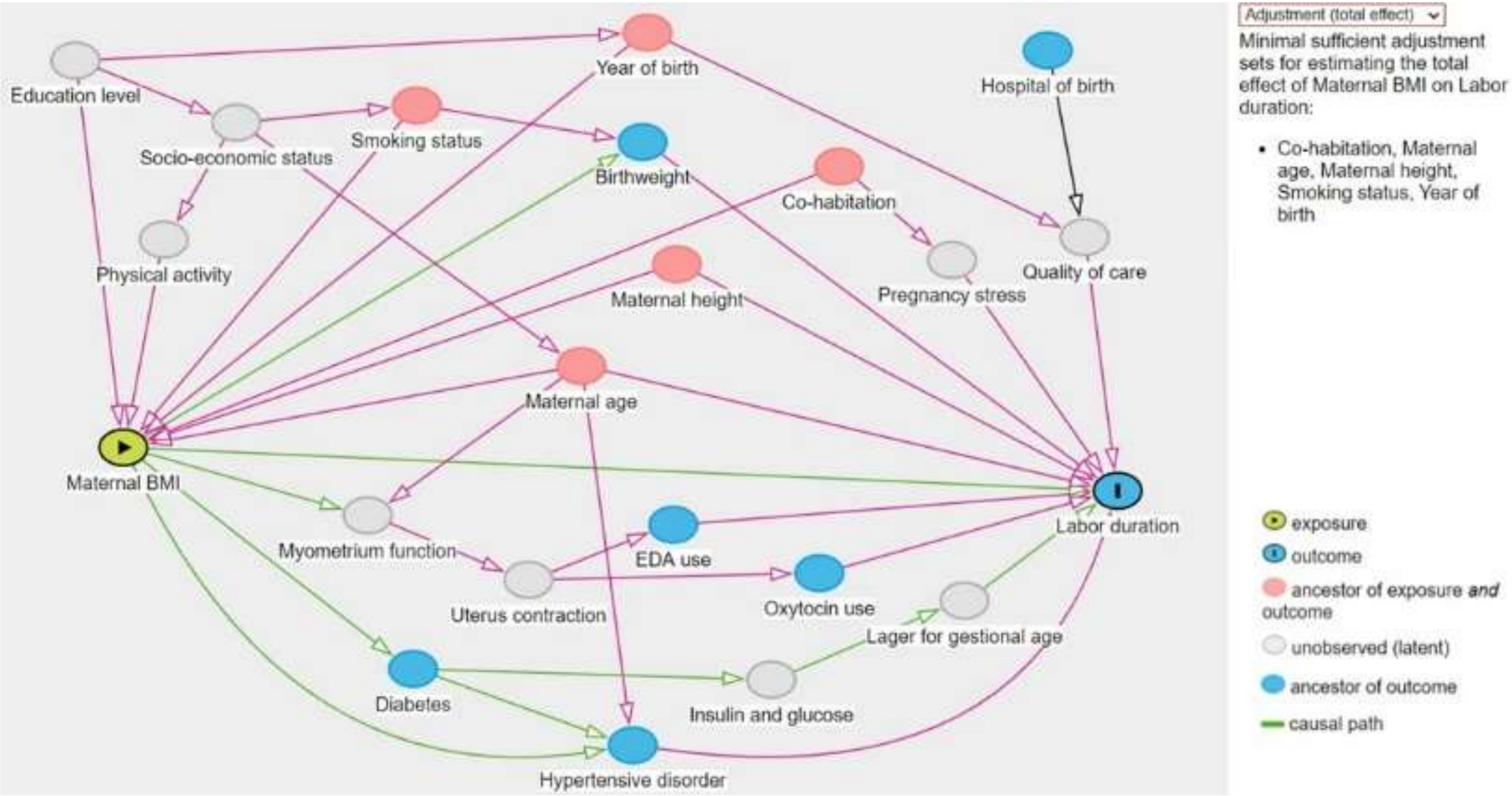

Suppl. Fig 1. Identification of confounders and mediators. Illustration of pathways from maternal BMI to duration of labor through the directed acyclic gram by DAGitty.

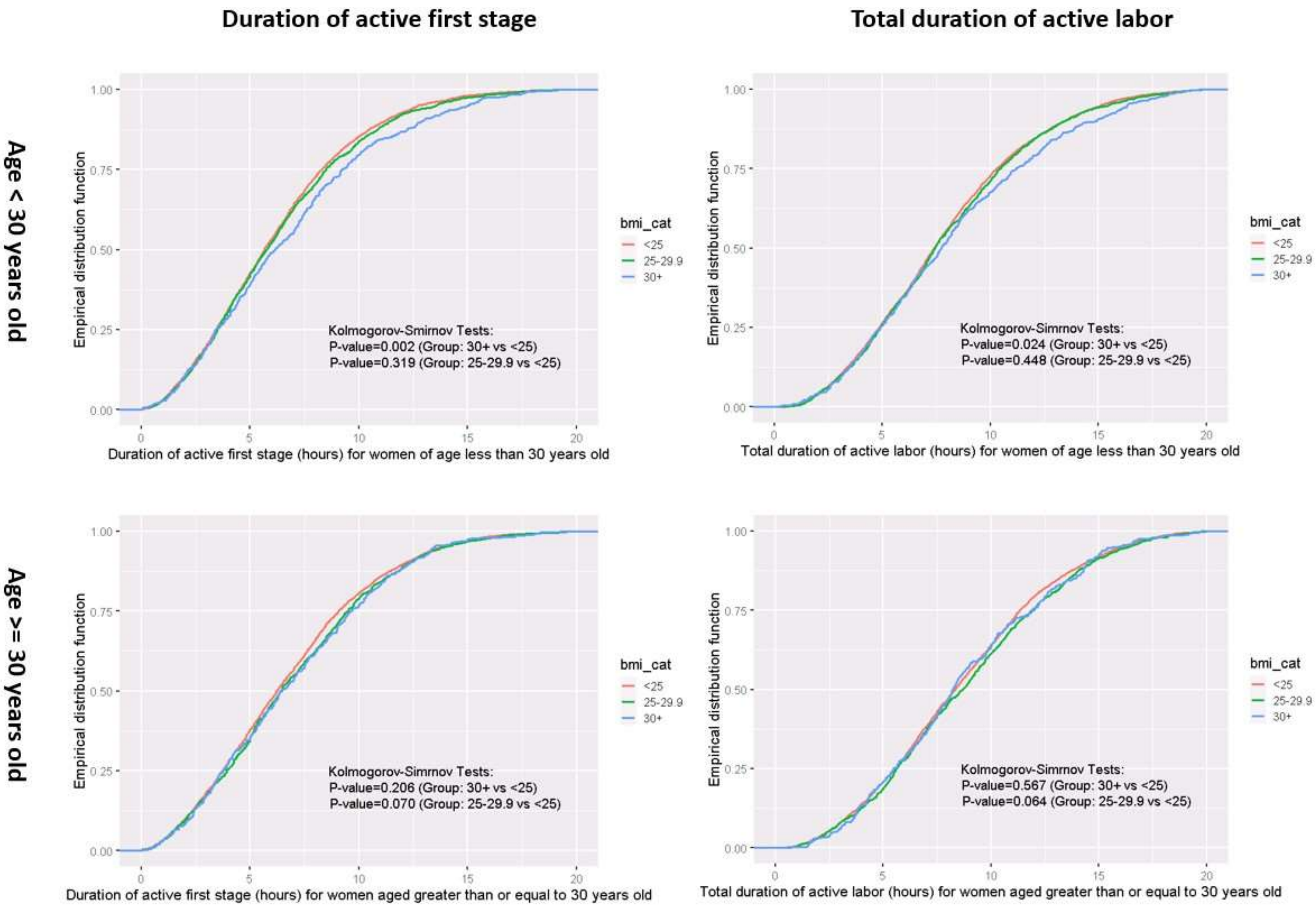
Duration of active first stage
Total duration of active labor
Age < 30 years old
Age >= 30 years old
Empirical distribution function
bmi_cat
<25
25-29.9
30+
Kolmogorov-Smirnov Tests:
P-value=0.002 (Group: 30+ vs <25)
P-value=0.319 (Group: 25-29.9 vs <25)
Duration of active first stage (hours) for women of age less than 30 years old
Kolmogorov-Simrnov Tests:
P-value=0.024 (Group: 30+ vs <25)
P-value=0.448 (Group: 25-29.9 vs <25)
Total duration of active labor (hours) for women of age less than 30 years old
Kolmogorov-Simrnov Tests:
P-value=0.206 (Group: 30+ vs <25)
P-value=0.070 (Group: 25-29.9 vs <25)
Duration of active first stage (hours) for women aged greater than or equal to 30 years old
Kolmogorov-Simrnov Tests:
P-value=0.567 (Group: 30+ vs <25)
P-value=0.064 (Group: 25-29.9 vs <25)
Total duration of active labor (hours) for women aged greater than or equal to 30 years old

Suppl. Fig 2. Illustration of distributions of labour duration and comparisons between obese and normal-weight (or overweight vs normal-weight) using the KS-test, stratified by two age-group (i.e. < 30 vs ≥ 30 years old).

Suppl.Table 1. Multivariable regression analysis for association of duration of active first stage with maternal early-pregnancy BMI, using the Stockholm-Gotland obstetric database, 2008-2014.

	Maternal BMI (kg/m^2)	Multivariable regression estimates: **difference in duration of active first stage** [a] at mean or quantiles (95% confidence intervals, hours)									
		Linear regression	Quantile regression estimates								
		Mean	q10	q20	q30	q40	q50	q60	q70	q80	q90
Total study population	<25 (reference)	0	0	0	0	0	0	0	0	0	0
	25-29.9	0.13 (-0.04, 0.30)	0.07 (-0.11, 0.25)	0.10 (-0.08, 0.28)	0.12 (-0.06, 0.29)	0.02 (-0.16, 0.21)	0.08 (-0.14, 0.29)	0.16 (-0.06, 0.37)	0.25 (0.04, 0.47)	0.20 (-0.09, 0.49)	0.29 (-0.10, 0.68)
	≥30	0.43** (0.17, 0.70)	0.12 (-0.18, 0.41)	0.06 (-0.18, 0.30)	0.13 (-0.19, 0.44)	0.09 (-0.22, 0.40)	0.47* (0.04, 0.91)	0.61** (0.24, 0.97)	0.71** (0.40, 1.03)	**0.80** (0.36, 1.24)**	**0.85* (0.35, 1.35)**

multivariable regression analysis on the total study population: maternal early pregnancy BMI categorized into three sub-groups, with adjustment for maternal age and height (in restricted cubic splines with 3 degrees of freedom), smoking status (dummy variable), co-habitation status (categorical variable), year of birth (categorical variable).
[a]duration of active first stage: the length from the start of active phase of labour until the time point of the cervix fully dilated.

Suppl.Table 2. Multivariable regression analysis for association of **total duration of active labour** with maternal early-pregnancy BMI, using the Stockholm-Gotland obstetric database, 2008-2014

	Maternal BMI (kg/m^2)	Multivariable regression estimates: **difference in total duration of active labour** [a] at mean or quantiles (95% confidence intervals, hours)									
		Linear regression	Quantile regression estimates at below quantiles								
		Mean	q10	q20	q30	q40	q50	q60	q70	q80	q90
[a] Total study population	<25 (reference)	0	0	0	0	0	0	0	0	0	0
	25-29.9	0.15 (-0.03, 0.34)	0.11 (-0.12, 0.33)	0.15 (-0.05, 0.35)	0.05 (-0.16, 0.25)	0.15 (-0.08, 0.37)	0.15 (-0.11, 0.40)	0.26 (0.03, 0.49)	0.19 (-0.08, 0.45)	0.23 (-0.06, 0.51)	0.22 (-0.21, 0.65)
	30+	0.35* (0.05, 0.64)	0.18 (-0.11, 0.47)	0.05 (-0.30, 0.40)	0.11 (-0.27, 0.49)	0.08 (-0.30, 0.45)	0.09 (-0.34, 0.52)	0.36 (-0.04, 0.76)	**0.48* (0.04, 0.93)**	**0.71* (0.12, 1.29)**	**1.02** (0.55, 1.50)**

multivariable regression analysis on the whole study population: maternal early pregnancy BMI categorized into three sub-groups, with adjustment for maternal age and height (in restricted cubic splines with 3 degrees of freedom), smoking status (dummy variable), co-habitation status (categorical variable), year of birth (categorical variable).
a. total duration of active labour: the length from the time point of the cervix fully dilated until birth.

Suppl. Table 3. Crude regression analysis for association of **total duration of active labour** with maternal early-pregnancy BMI, using the Stockholm-Gotland obstetric database, 2008-2014

	Maternal BMI (kg/m^2)	Univariate regression estimates: **difference in total duration of active labour** [c]at mean or quantiles (95% confidence intervals, hours)									
		Linear regression	Quantile regression estimates at below quantiles								
		Mean	q10	q20	q30	q40	q50	q60	q70	q80	q90
[a] Total study population	<25 (reference)	0	0	0	0	0	0	0	0	0	0
	25-29.9	0.19<br>(0.00, 0.37)	0.07<br>(-0.19, 0.32)	0.13<br>(-0.07, 0.34)	0.03<br>(-0.20, 0.27)	0.13<br>(-0.07, 0.34)	0.12<br>(-0.16, 0.39)	0.25<br>(0.00, 0.50)	0.25<br>(-0.02, 0.52)	0.35<br>(0.01, 0.69)	0.23<br>(-0.15, 0.62)
	30+	0.30*<br>(0.00, 0.60)	0.18<br>(-0.13, 0.50)	-0.05<br>(-0.38, 0.28)	-0.02<br>(-0.37, 0.34)	0.18<br>(-0.25, 0.61)	0.2<br>(-0.14, 0.54)	**0.12**<br>(-0.42, 0.65)	**0.47**<br>(-0.05, 0.99)	**0.78****<br>(0.31, 1.25)	**0.78***<br>(0.11, 1.46)
[b] Younger age group (<30)	<25 (reference)	0	0	0	0	0	0	0	0	0	0
	25-29.9	0.16<br>(-0.08, 0.41)	0.07<br>(-0.21, 0.35)	0.13<br>(-0.11, 0.38)	0.08<br>(-0.21, 0.38)	0.13<br>(-0.15, 0.42)	0.05<br>(-0.24, 0.34)	0.23<br>(-0.13, 0.60)	0.32<br>(-0.02, 0.65)	0.2<br>(-0.22, 0.62)	0.13<br>(-0.41, 0.68)
	30+	0.54**<br>(0.16, 0.92)	0.13<br>(-0.18, 0.45)	0.13<br>(-0.24, 0.51)	0.18<br>(-0.29, 0.65)	0.05<br>(-0.44, 0.54)	0.37<br>(-0.10, 0.84)	0.42<br>(-0.20, 1.03)	**0.86****<br>(0.22, 1.51)	**1.25****<br>(0.58, 1.92)	**1.67****<br>(0.84, 2.49)
[b] Older age group (>=30)	<25 (reference)	0	0	0	0	0	0	0	0	0	0
	25-29.9	0.26<br>(-0.02, 0.53)	0.22<br>(-0.18, 0.61)	0.27<br>(-0.04, 0.57)	0.1<br>(-0.25, 0.45)	0.13<br>(-0.23, 0.50)	0.38<br>(-0.03, 0.80)	0.35<br>(-0.04, 0.74)	0.35<br>(-0.09, 0.79)	0.73**<br>(0.27, 1.20)	0.2<br>(-0.36, 0.76)
	30+	0.12<br>(-0.36, 0.59)	0.28<br>(-0.01, 0.58)	-0.02<br>(-0.63, 0.59)	0.1<br>(-0.55, 0.75)	0.17<br>(-0.40, 0.74)	-0.08<br>(-0.56, 0.39)	0.13<br>(-0.61, 0.87)	0.05<br>(-0.90, 1.00)	0.43<br>(-0.48, 1.34)	-0.03<br>(-0.68, 0.62)

a: Crude (or Univariate) regression analysis on the total study population, without adjusting for any covariates.

b: Crude (or Univariate) regression analysis on two age-stratified sub-population separately, without adjusting for any covariates.

c: total duration of active labour: the length from the time point of the cervix fully dilated until birth.

* P-value < 0.05; **P-value < 0.01

Suppl. Table 4. Crude regression analysis for association of **duration of active first stage** with maternal early-pregnancy BMI, using the Stockholm-Gotland obstetric database, 2008-2014

	Maternal BMI (kg/m^2)	Univariate regression estimates: **difference in duration of active first stage** [c] at mean or quantiles (95% confidence intervals, hours)									
		Linear regression	Quantile regression estimates at below quantiles								
		Mean	q10	q20	q30	q40	q50	q60	q70	q80	q90
[a] Total study population	<25 (reference)	0	0	0	0	0	0	0	0	0	0
	25-29.9	0.17* (0.01, 0.34)	0.05 (-0.11, 0.21)	0.08 (-0.10, 0.26)	0.12 (-0.07, 0.30)	0.1 (-0.08, 0.28)	0.13 (-0.07, 0.34)	0.15 (-0.11, 0.41)	0.28 (0.05, 0.52)	0.33 (0.06, 0.61)	0.23 (-0.11, 0.58)
	30+	0.42** (0.16, 0.69)	0.08 (-0.13, 0.30)	0.08 (-0.17, 0.33)	0.12 (-0.21, 0.44)	0.2 (-0.13, 0.53)	0.37 (-0.11, 0.85)	0.50** (0.14, 0.86)	0.72** (0.29, 1.14)	0.75** (0.30, 1.204)	0.93** (0.40, 1.47)
[b] Younger age group (<30)	<25 (reference)	0	0	0	0	0	0	0	0	0	0
	25-29.9	0.18 (-0.04, 0.40)	0.1 (-0.14, 0.34)	0.05 (-0.19, 0.29)	0.03 (-0.18, 0.25)	0.05 (-0.23, 0.33)	0.13 (-0.16, 0.42)	0.08 (-0.20, 0.36)	0.28 (-0.03, 0.59)	0.42 (-0.02, 0.86)	0.32 (-0.14, 0.77)
	30+	0.61** (0.27, 0.95)	0.02 (-0.25, 0.28)	0.05 (-0.27, 0.37)	0.18 (-0.32, 0.69)	0.3 (-0.13, 0.73)	0.5 (-0.12, 1.12)	0.73** (0.34, 1.13)	0.82** (0.23, 1.41)	1.00** (0.37, 1.64)	1.77** (0.93, 2.60)
[b] Older age group (>=30)	<25 (reference)	0	0	0	0	0	0	0	0	0	0
	25-29.9	0.2 (-0.06, 0.45)	0.07 (-0.16, 0.30)	0.08 (-0.23, 0.40)	0.25 (-0.07, 0.57)	0.1 (-0.18, 0.38)	0.2 (-0.16, 0.56)	0.25 (-0.12, 0.62)	0.48** (0.16, 0.81)	0.2 (-0.24, 0.64)	0.13 (-0.32, 0.59)
	30+	0.25 (-0.18, 0.68)	0.23 (-0.15, 0.61)	0.07 (-0.35, 0.49)	0 (-0.54, 0.54)	0.23 (-0.30, 0.76)	0.17 (-0.42, 0.75)	0.3 (-0.39, 0.99)	0.58 (-0.03, 1.20)	0.5 (-0.16, 1.16)	0.23 (-0.63, 1.10)

a: Crude (or Univariate) regression analysis on the total study population, without adjusting for any covariates.
b: Crude (or Univariate) regression analysis on two age-stratified sub-population separately, without adjusting for any covariates.
c: duration of active first stage: the length from the start of active phase of labour until the time point of the cervix fully dilated.
* P-value < 0.05; **P-value < 0.01

Suppl. Table 5: Baseline characteristic for both the Study population and Target-population for comparison of study characteristics

	Study population (n=13,794)	Target population (located in Robson Group 1) dilation at admission ≤ 5 cm (n=39,576)	dilation at admission any cm (n=52,754)
**Mother's characteristic:**			
**Age, years, mean (SD)**	29.1 (5.0)	29.2 (4.9)	29.3 (4.9)
**Early-pregnancy BMI, kg/m^2**, mean (SD)	23.5 (4.1)	23.4 (3.9)	23.2 (3.9)
**Maternal height, cm, mean (SD)**	166.0 (6.5)	166.3 (6.5)	166.6 (6.5)
**Gestational age in days,** *mean (SD)*	281.5 (7.5)	281.2 (7.6)	280.8 (7.7)
**Family situation**			
Single	6,24%	6,25%	6,03%
Co-habitant	90,22%	90,35%	90,73%
Not known	3,54%	3,40%	3,23%
**Smoking status**			
Non-smoker	94,45%	94,67%	94,95%
Smoker	4,84%	4,52%	4,24%
Not known	0,71%	0,81%	0,81%
**Diabetes**	0,49%	0,45%	0,42%
**Hypertension**	3,94%	3,76%	3,62%
**Characteristics related to labour:**			
**Oxytocin augmentation**	52,09%	43,30%	35,47%
**Epidural analgesia**	66,29%	60,79%	51,20%
**Mode of birth**			
Spontaneous vaginal	72,50%	73,83%	76,63%
Operative vaginal	17,70%	16,52%	15,50%
Cesarean section	9,81%	9,66%	7,87%
**Duration of second stage in mins, median (IQR)**	93 (50-162)	92 (50-162)	90 (49-159)
**Fetal position at birth**			
Anterior	94,01%	94,31%	94,75%
Posterior	5,99%	5,69%	5,25%
**Infant's characteristics:**			
**Birthweight (gram), mean (SD)**	3506 (442)	3498 (441)	3488 (438)
**Year of birth**			
2008-2010	38,05%	39,53%	39,64%
2011-2014	61,95%	60,47%	60,36%

Suppl. Table 6. A complementary analysis of association between **total duration of active labour** and maternal early-pregnancy BMI, **excluding women with C-section in second stage**

	Maternal BMI (kg/m^2)	Multivariable regression estimates: **difference in total duration of active labour** [c] at mean or quantiles (95% confidence intervals, hours)									
		Linear regression	Quantile regression estimates at below quantiles								
		Mean	q10	q20	q30	q40	q50	q60	q70	q80	q90
[a] Total study population	<25 (reference)	0	0	0	0	0	0	0	0	0	0
	25-29.9	0.11 (-0.07, 0.30)	0.09 (-0.13, 0.31)	0.18 (-0.03, 0.38)	0.05 (-0.16, 0.27)	0.10 (-0.14, 0.34)	0.11 (-0.14, 0.36)	0.21 (-0.04, 0.45)	0.13 (-0.11, 0.45)	0.15 (-0.14, 0.44)	0.09 (-0.31, 0.49)
	30+	0.25 (-0.05, 0.55)	0.17 (-0.11, 0.45)	0.12 (-0.48, 0.24)	-0.15 (-0.52, 0.20)	0.06 (-0.45, 0.33)	0.12 (-0.30, 0.54)	0.28 (-0.13, 0.70)	0.40 (-0.02, 0.81)	0.49 (-0.09, 1.07)	**0.91* (0.19, 1.63)**
[b] Younger age group (<30)	<25 (reference)	0	0	0	0	0	0	0	0	0	0
	25-29.9	0.05 (-0.19, 0.29)	0.05 (-0.20, 0.30)	0.05 (-0.21, 0.31)	-0.03 (-0.34, 0.28)	0.11 (-0.20, 0.42)	0.00 (-0.28, 0.28)	0.15 (-0.22, 0.51)	0.16 (-0.14, 0.46)	0.02 (-0.36, 0.39)	-0.02 (-0.50, 0.46)
	30+	0.34 (-0.03, 0.72)	0.00 (-0.35, 0.35)	-0.09 (-0.59, 0.41)	-0.13 (-0.55, 0.30)	-0.10 (-0.59, 0.39)	0.38 (-0.21, 0.97)	0.35 (-0.12, 0.81)	0.45 (-0.16, 1.06)	0.58 (-0.23, 1.39)	**1.65** (0.90, 2.40)**
[b] Age-stratified analysis (on the older age group, >=30 y)	<25 (reference)	0	0	0	0	0	0	0	0	0	0
	25-29.9	0.18 (-0.11, 0.46)	0.04 (-0.33, 0.42)	0.30 (-0.04, 0.64)	0.08 (-0.24, 0.40)	0.06 (-0.28, 0.39)	0.20 (-0.19, 0.59)	0.16 (-0.26, 0.59)	0.29 (-0.08, 0.66)	0.32 (-0.14, 0.78)	0.39 (-0.22, 1.00)
	30+	0.09 (-0.40, 0.58)	0.15 (-0.28, 0.58)	-0.02 (-0.54, 0.50)	-0.40 (-1.07, 0.27)	-0.14 (-0.75, 0.48)	-0.02 (-0.68, 0.65)	0.06 (-0.74, 0.87)	0.25 (-0.58, 1.09)	0.62 (-0.13, 1.37)	0.50 (-0.18, 1.18)

a: multivariable regression analysis on the whole study population: maternal early pregnancy BMI categorized into three sub-groups, with adjustment for maternal age and height (in restricted cubic splines with 3 degrees of freedom), smoking status (dummy variable), co-habitation status (categorical variable), year of birth (categorical variable).

b: multivariable regression analysis on two age sub-groups separately: maternal early pregnancy BMI categorized into three sub-groups, with adjustment for maternal height (in restricted cubic splines with 3 degrees of freedom), smoking status (dummy variable), co-habitation status (categorical variable), year of birth (categorical variable); stratified on two separate age groups.

c: total duration of active labour: the length from the time point of the cervix fully dilated until birth.

* P-value < 0.05; **P-value < 0.01
